# Loss of Integrase Interactor 1 (INI1) Expression in a Subset of Differentiated Thyroid Cancer

**DOI:** 10.3390/diagnostics10050280

**Published:** 2020-05-05

**Authors:** Kung-Chen Ho, Jie-Jen Lee, Chi-Hsin Lin, Ching-Hsiang Leung, Shih-Ping Cheng

**Affiliations:** 1 Department of Surgery, MacKay Memorial Hospital and Mackay Medical College, Taipei 104215, Taiwan; kchoox@yahoo.com.tw (K.-C.H.); leejj1957@pchome.com.tw (J.-J.L.); 2 Department of Medical Research, MacKay Memorial Hospital, Taipei 104215, Taiwan; chihsin@outlook.com; 3 Department of Bioscience Technology, Chung Yuan Christian University, Taoyuan City 320314, Taiwan; 4 Division of Endocrinology and Metabolism, Department of Internal Medicine, MacKay Memorial Hospital and Mackay Medical College, Taipei 104215, Taiwan; louisapoupou@hotmail.com.tw; 5 Department of Pharmacology, School of Medicine, College of Medicine, Taipei Medical University, Taipei 110301, Taiwan

**Keywords:** SMARCB1, integrase interactor 1, thyroid carcinoma, aneuploidy

## Abstract

Alterations in the switching defective/sucrose non-fermenting (SWI/SNF) chromatin-remodeling complex are enriched in advanced thyroid cancer. Integrase interactor 1 (INI1), encoded by the *SMARCB1* gene on the long arm of chromosome 22, is one of the core subunits of the SWI/SNF complex. INI1 immunohistochemistry is frequently used for the diagnosis of malignant rhabdoid neoplasms. In the present study, we found normal and benign thyroid tissues generally had diffusely intense nuclear immunostaining. Loss of INI1 immunohistochemical expression was observed in 8% of papillary thyroid cancer and 30% of follicular thyroid cancer. Furthermore, loss of INI1 expression was associated with extrathyroidal extension (*p* < 0.001) and lymph node metastasis (*p* = 0.038). Analysis of The Cancer Genome Atlas database revealed that SMARCB1 underexpression was associated with the follicular variant subtype and aneuploidy in papillary thyroid cancer. We speculate that SMARCB1 is an important effector in addition to NF2 and CHEK2 inactivation among thyroid cancers with chromosome 22q loss.

## 1. Introduction

Thyroid cancer has a wide spectrum of different morphologies and clinical behaviors, ranging from indolent differentiated thyroid cancer to aggressive and invariably lethal anaplastic cancer. Poorly differentiated thyroid cancer takes an intermediate position on the progression spectrum. Along the process of dedifferentiation, activation of the phosphatidylinositol 3-kinase (PI3K)-AKT pathway has long been recognized as a key molecular event [1]. Recently, alterations in switching defective/sucrose non-fermenting (SWI/SNF) complex, mismatch repair genes, and histone methyltransferases were found as novel mechanisms and therapeutic targets in advanced thyroid cancer [2].

SWI/SNF chromatin-remodeling complex consists of multiple subunits and regulates transcriptional activity by binding to promoters or other regulatory regions. Depending on whether a transcriptional activator or repressor recruits SWI/SNF, transcription can be upregulated or downregulated. Human SWI/SNF complex contains a single ATPase, either BRM (encoded by the *SMARCA2* gene) or BRG1 (*SMARCA4*), and three main core subunits: BAF155 (*SMARCC1*), BAF170 (*SMARCC2*), and BAF47 (*SMARCB1*) [3]. SMARCB1, a human homolog of yeast transcription factor SNF5, was isolated through its interaction with human immunodeficiency virus integrase and is also named INI1 (integrase interactor 1) [4]. Versteege and colleagues firstly reported deletions and loss-of-function mutations in the *hSNF5/INI1* gene in malignant pediatric rhabdoid tumors [5]. Thereafter, the INI1 expression pattern has been frequently used by pathologists for the diagnosis of malignant rhabdoid tumors.

Loss of INI1 expression has been further identified in a variety of other malignant neoplasms [6]. Considering that alterations in the SWI/SNF chromatin-remodeling complex may provide prognostic implications in thyroid carcinogenesis, the aim of the present study was to evaluate the expression of INI1 and its clinicopathological relevance in differentiated thyroid cancer.

## 2. Materials and Methods

### 2.1. Study Population

This study (12MMHIS149; valid from 14 December 2012 to 13 December 2021) was approved and monitored by the Institutional Review Board of MacKay Memorial Hospital. Patients who underwent thyroidectomy for benign or malignant thyroid disease were de-identified and randomly selected [7]. Sections of formalin-fixed and paraffin-embedded tissue samples from pathology department archives were subjected to immunohistochemical staining.

### 2.2. Immunohistochemistry

Tissue sections were deparaffinized and rehydrated, followed by microwave-based antigen retrieval in citrate buffer [8]. Immunostaining for INI1 was performed with a commercially available monoclonal antibody clone 25 (Zeta Corporation, Arcadia, CA, USA). Detection of INI1 expression was performed using MACH 4 Universal HRP-Polymer (Biocare Medical, Pacheco, CA, USA), followed by incubation with 3,3’-diaminobenzidine (DAB) (Dako-Agilent Technologies, Glostrup, Denmark) and counterstaining with hematoxylin. Negative controls were performed by omitting the primary antibody.

### 2.3. Interpretation of INI1 Staining

Two independent investigators blinded for clinical data evaluated the nuclear INI1 immunostaining. Disagreements were resolved by discussion, or a third expert was asked to arbitrate. The staining intensity was scored as negative, weak, moderate, or strong [9]. Given that normal and benign thyroid tissues generally had diffusely intense immunostaining, malignant thyroid tumors exhibiting strong or moderate nuclear staining were considered as INI1-intact. Those exhibiting weak INI1 staining were considered as INI1-loss in the presence of positive internal control.

### 2.4. Analysis of Publicly Available Genomics Dataset

We accessed the public functional genomics data repository, Gene Expression Omnibus (GEO), at the National Center for Biotechnology Information. GSE6004 comprises gene expression data of seven paired central and invasion regions of papillary thyroid cancer, as well as four normal tissues [10]. Expression profiling was performed using the Affymetrix Human Genome U133 Plus 2.0 microarray platform (Affymetrix; Thermo Fisher Scientific, Santa Clara, CA, USA). Reported somatic mutations of the *SMARCB1* gene were explored using the Catalogue of Somatic Mutations in Cancer (COSMIC) at the Wellcome Sanger Institute [11].

### 2.5. Analysis of The Cancer Genome Atlas (TCGA)

RNA-seq expression data and somatic copy number alterations were downloaded from the thyroid cancer (THCA) database of TCGA, as we previously reported [12,13,14]. Cases with unknown status of the extrathyroidal extension were excluded from the analysis. The expression level was quantified as RNA-Seq by Expectation Maximization (RSEM). A *Z*-score was calculated to reflect the degree of variation in gene expression. Underexpression was defined as a *Z*-score < −2, and overexpression was defined as a *Z*-score > 2. Correlations of gene expression levels were analyzed using the Gene Expression Profiling Interactive Analysis (GEPIA) database [15].

### 2.6. Statistical Analysis

Differences between categorical variables were assessed using Fisher’s exact probability test. For ordered groups, comparisons were done using the Cochran–Armitage trend test for categorical variables and the Jonckheere–Terpstra test for continuous variables [16]. Cohen’s κ coefficient was used to measure the inter-rater agreement [17]. All statistical tests were two-sided at the 5% significance level and were performed using STATA statistical software version 14.0 (StataCorp, College Station, TX, USA).

## 3. Results

**Figure 1:**
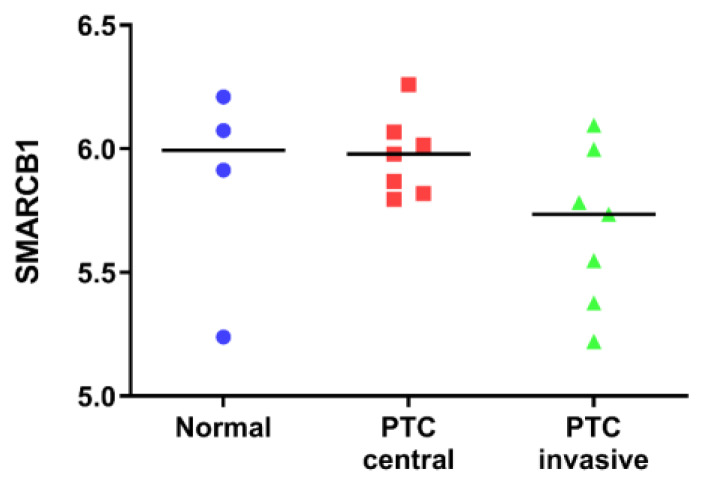
SMARCB1 gene expression levels from a published dataset (GSE6004) in normal thyroid tissues, tumor centers of papillary thyroid cancer (PTC), and invasive areas of PTC. Data are reported as scatter plots of log_2_ values and medians.

To preliminarily assess whether SMARCB1 was differentially expressed in thyroid cancer, we examined a microarray dataset downloaded from GEO (series number GSE6004). We found that SMARCB1 appeared downregulated in the invasive part of papillary thyroid cancer, while the tumor center retained the same expression level as normal thyroid tissues (Figure 1).

**Figure 2:**
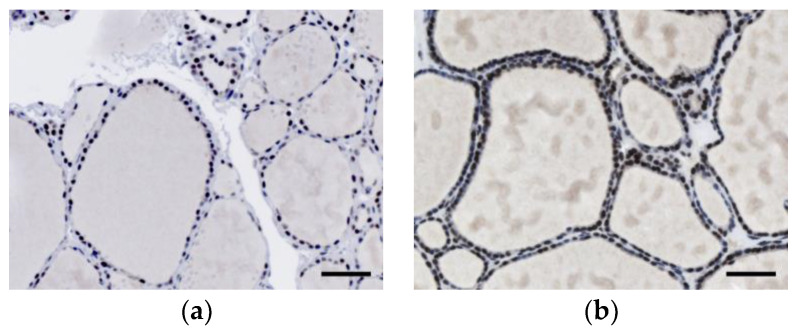

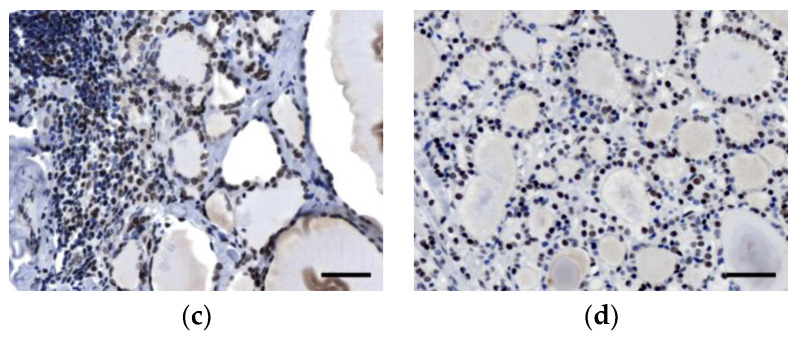
Immunohistochemical expression of integrase interactor 1 (INI1) in (**a**) normal thyroid tissue, (**b**) nodular goiter, (**c**) lymphocytic thyroiditis, and (**d**) follicular adenoma. Scale bars: 50 μm.

We next analyzed the immunohistochemical expression and localization of INI1 in normal thyroid tissue (*n* = 10), nodular goiter (*n* = 10), lymphocytic thyroiditis (*n* = 5), and follicular adenoma (*n* = 10). As shown in Figure 2, strong staining was observed in the nucleus of normal and benign thyroid tissues. Focal loss of expression was seen in some epithelial cells of follicular adenoma. Nonetheless, more than half of the cells retained the intact INI1 expression.

**Figure 3:**
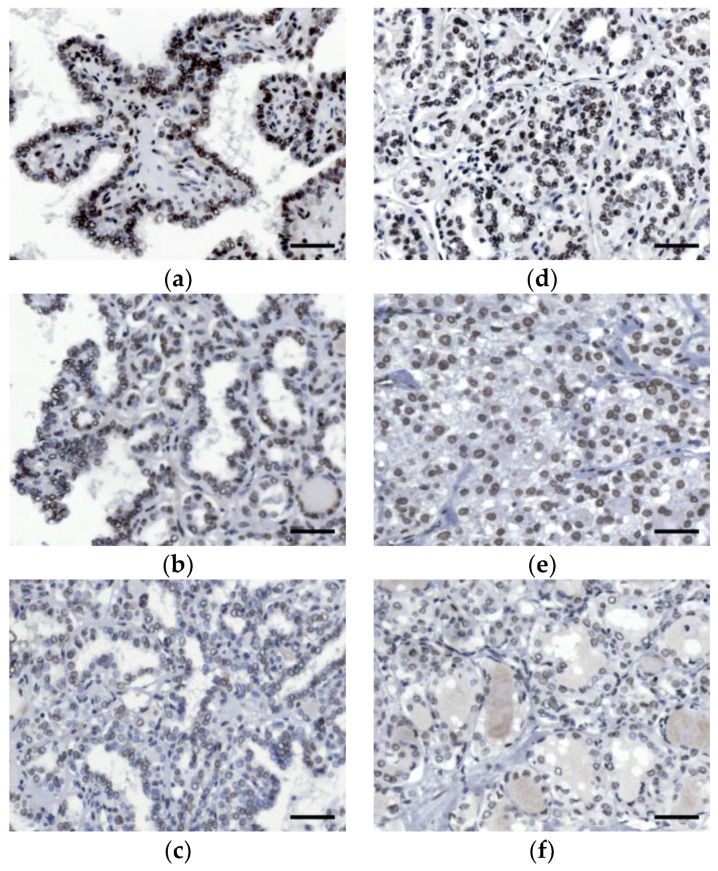
Immunohistochemical expression of integrase interactor 1 (INI1) in (**a**–**c**) papillary thyroid cancer and (**d**–**f**) follicular thyroid cancer. Representative microphotographs of (**a**,**d**) strong, (**b**,**e**) moderate, and (**c**,**f**) weak nuclear expression are shown. Scale bars: 50 μm.

A total of 63 cases of differentiated thyroid cancer were further analyzed. No tumor we examined was completely negative for INI1 staining. However, some of the cases demonstrated decreased nuclear staining and were classified as moderate or weak expression. The κ agreement score was 0.714 (95% confidence interval: 0.429 to 0.924), indicating a substantial agreement. Representative cases of differentiated thyroid cancer expressing varying levels of INI1 staining are depicted in Figure 3.

For statistical purposes, cases with weak staining were defined as loss of INI1 expression. Accordingly, 8% of papillary thyroid cancer cases and 30% of follicular thyroid cancer cases had a loss of INI1 expression. The clinicopathological characteristics of INI1-intact and INI1-loss patients are summarized in Table 1. Loss of INI1 expression was significantly associated with the occurrence of extrathyroidal extension and lymph node metastasis in differentiated thyroid cancer.

To corroborate our findings, the RNA-seq expression data of SMARCB1 were obtained from the THCA database of TCGA. In all, 485 patients with papillary thyroid cancer had sufficient information for the analysis. After applying the *Z*-score transformation, 47 (10%) patients had SMARCB1 underexpression, and 20 (4%) had SMARCB1 overexpression. Papillary thyroid cancer with SMARCB1 underexpression was more likely to be the follicular variant (Table 2). Nonetheless, the trend for extrathyroidal extension or lymph node metastasis did not reach statistical significance.

We found 16 *SMARCB1* mutation descriptions out of 1328 tested thyroid cancer samples in COSMIC. The histology type of most cases with somatic *SMARCB1* mutations was anaplastic thyroid cancer. Only one deletion/frameshift mutation (c.1175del, p.P392Rfs*100) was reported in papillary thyroid cancer.

**Figure 4:**
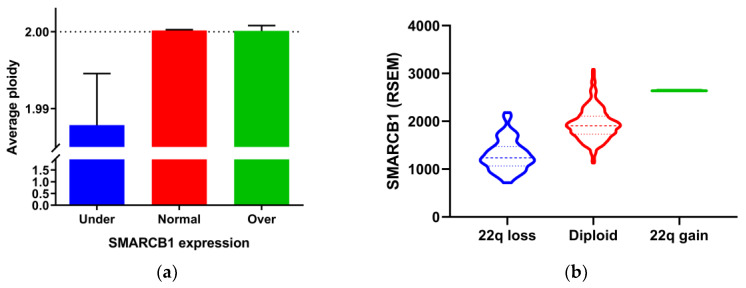
Associations between SMARCB1 gene expression and genetic alterations in papillary thyroid cancer (THCA) dataset from The Cancer Genome Atlas (TCGA). (**a**) Bar graph showing the median ploidy of tumors with underexpression, normal expression, and overexpression of SMARCB1. Error bars indicate interquartile range. (**b**) Violin plot showing SMARCB1 expression levels grouped by somatic copy number alterations of chromosome 22q. RSEM: RNA-Seq by Expectation Maximization.

Interestingly, we noted that tumors with SMARCB1 underexpression had a significantly lower ploidy (Figure 4a). The *SMARCB1* gene maps to the long arm of chromosome 22 at position 11.23 (22q11.23). It is worth noting that the most common somatic copy number alteration in papillary thyroid cancer is characterized as chromosome 22q loss [18]. In our analysis, the majority (89%) of cases with SMARCB1 underexpression had chromosome 22q loss. We further explored the relationship between the SMARCB1 expression level and copy number alterations of chromosome 22q. As expected, chromosome 22q loss was significantly associated with a lower expression level of SMARCB1 (Figure 4b).

**Figure 5:**
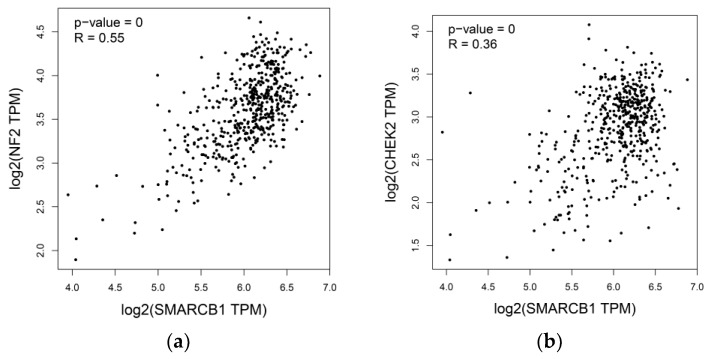
Associations of SMARCB1 expression levels and the expression of (**a**) NF2 and (**b**) CHEK2 in papillary thyroid cancer (THCA) dataset from The Cancer Genome Atlas (TCGA). These three genes are all located on chromosome 22q. TPM: transcripts per million.

It has been advocated that chromosome 22q loss is associated with loss of NF2 and CHEK2 tumor suppressors and is influential in the carcinogenesis of papillary thyroid cancer [19]. We mined TCGA data using the GEPIA database to investigate the relationship between SMARCB1 and NF2/CHEK2 expression. As shown in Figure 5, a significantly positive correlation was present between the expression levels of SMARCB1 and NF2, as well as SMARCB1 and CHEK2. Taken together, these results suggest that chromosome 22q loss might play an important role in determining the expression level of these tumor suppressor genes.

## 4. Discussion

SWI/SNF chromatin-remodeling complex controls fundamental cellular processes by promoting or repressing the expression of specific genes. Mutations in genes encoding SWI/SNF subunits are observed in a large variety of human cancers [20]. In a landmark study, subunits of the SWI/SNF complex were found to be mutated in 36% of anaplastic thyroid cancer cases and 6% of poorly differentiated cancer cases [21]. Subsequent studies confirmed that genes encoding components of the SWI/SNF chromatin-remodeling complex were mutated in 9% to 16% of advanced thyroid cancer cases [22,23,24]. In general, these mutations were mutually exclusive. Genetic complexity is also recapitulated in human thyroid cancer cell lines, as 31% of thyroid cancer cell lines harbor genetic alterations in members of the SWI/SNF complex [25].

SMARCB1 is one of the core subunits of the SWI/SNF complex. Germline mutations in *SMARCB1* are responsible for rhabdoid tumor predisposition syndrome and familial schwannomatosis [26]. Currently, *SMARCB1* is classified into Tier 1 of the Cancer Gene Census as a tumor suppressor gene [27]. Although it was reported that *SMARCB1* mutations were acquired in about 6% of anaplastic thyroid cancer cases [21], somatic mutation of the *SMARCB1* gene is very uncommon in differentiated thyroid cancer. Of 57 patients with fatal non-anaplastic thyroid cancer, mutations in *SMARCB1* were identified in 4% of the cohort [22]. Recently, one of 35 patients with follicular thyroid cancer was found to have the *SMARCB1* mutation [28]. In this regard, we propose that chromosome 22q loss, instead of somatic mutation, plays a major role in the loss of INI1 expression in differentiated thyroid cancer.

It is well known that aneuploidy is more common in the follicular variant of papillary thyroid cancer and follicular thyroid cancer than classic papillary thyroid cancer [29]. Somatic copy number alterations are enriched in thyroid cancers lacking driver mutations or fusions, suggesting that these alterations are oncogenic driver events. Although chromosome 22q loss is the most common copy number alteration in papillary thyroid cancer [19], this copy number change is more often seen in follicular thyroid cancer [30]. In agreement with prior observations, we found that follicular cancer tended to have a higher frequency of loss of INI1 expression (*p* = 0.073) in the present study, and the follicular variant of papillary thyroid cancer was significantly associated with SMARCB1 underexpression in the TCGA dataset. In the meantime, SMARCB1 underexpression was more likely linked to a hypodiploid tumor genome.

INI1 immunohistochemistry has been established as a sensitive and specific tool for detecting and classifying malignant rhabdoid neoplasms. More and more benign and malignant tumors are being found to have a loss of nuclear INI1 expression, and the list of so-called SMARCB1-deficient neoplasms is expanding [31]. In the present study, we, for the first time, demonstrated that INI1 expression was lost in a subset of differentiated thyroid cancer. Importantly, the tumors with loss of INI1 expression appeared to be associated with a more aggressive phenotype, namely, extrathyroidal extension and lymph node metastasis. In light of SMARCB1 being a bona fide tumor suppressor, it is not surprising that loss of INI1 expression has been identified as a negative prognostic factor in other malignancies. For instance, loss of INI1 expression in patients with colorectal cancer was associated with shorter overall survival [32]. Recently, Agarwal et al. reported a case of thyroid cancer with negative INI1 expression and unique morphological features similar to that of INI1-deficient sinonasal carcinomas [33]. The patient died of widespread metastasis within two years after diagnosis. In this regard, loss of INI1 expression might have both prognostic and predictive significance. Although the frequencies of SWI/SNF mutations were similar between primary thyroid tumors and distant metastases, mutations in the SWI/SNF complex were associated with non-avidity of radioactive iodine therapy [24].

Somatic copy number alterations often encompass many genes. Inactivating NF2 and CHEK2 have been regarded as the main effectors of chromosome 22q loss [19]. Chromosome 22q loss was associated with a widely invasive type of follicular thyroid cancer [30], and NF2 loss promotes RAS-induced tumorigenesis [34]. Here, we propose that SMARCB1 underexpression also plays a critical role in the oncogenic effects of 22q loss. Studies have shown that SMARCB1 inactivation causes elevated levels of histone H3 lysine 27 trimethylation (H3K27me3) at lineage-specific Polycomb targets and confers susceptibility to EZH2 inhibitors [35,36]. We recently demonstrated that H3K27me3 overexpression was associated with aggressiveness and dedifferentiation in thyroid cancer [37]. Additional pathways associated with SMARCB1 inactivation include the p16-RB pathway, Wnt/β-Catenin pathway, and Sonic hedgehog signaling pathway [38]. These mechanisms might cooperate with NF2 and CHEK2 inactivation to mediate malignant behavior in thyroid cancer with chromosome 22q loss.

In conclusion, we found that a subset of differentiated thyroid cancer had loss of nuclear INI1 immunohistochemical expression, and we propose that loss of INI1 expression may be associated with a more aggressive phenotype. Underexpression of SMARCB1 is correlated with aneuploidy. We speculate that SMARCB1 is an important effector in addition to NF2 and CHEK2 inactivation among thyroid cancers with chromosome 22q loss.

## Figures and Tables

**Table 1:** Associations between clinicopathological features and the immunohistochemical expression of integrase interactor 1 (INI1) in differentiated thyroid cancer.

		All	INI1 Intact	INI1 Loss	*p*-Value
		(*n* = 63)	(*n* = 56)	(*n* = 7)	
Age					0.095
	<55 years	45 (71%)	42 (75%)	3 (43%)	
	≥55 years	18 (29%)	14 (25%)	4 (57%)	
Sex					0.646
	Male	14 (22%)	12 (21%)	2 (29%)	
	Female	49 (78%)	44 (79%)	5 (71%)	
Type					0.073
	Papillary	53 (84%)	49 (88%)	4 (57%)	
	Follicular	10 (16%)	7 (13%)	3 (43%)	
Extrathyroidal extension					<0.001
	No	48 (76%)	47 (84%)	1 (14%)	
	Yes	15 (24%)	9 (16%)	6 (86%)	
Lymph node metastasis					0.038
	No	35 (56%)	34 (61%)	1 (14%)	
	Yes	28 (44%)	22 (39%)	6 (86%)	

**Table 2:** Clinicopathological features of 485 patients with papillary thyroid cancer (THCA) from The Cancer Genome Atlas (TCGA) database stratified by the expression of SMARCB1. RSEM: RNA-Seq by Expectation Maximization.

		Under	Normal	Over	*p*-Value
		(*n* = 47)	(*n* = 418)	(*n* = 20)	
Expression (RSEM)		1119 (982–1208)	1879 (1682–2070)	2775 (2653–2921)	<0.001
Age (years)		51 (37–62)	46 (34–58)	51 (36–57)	0.573
Sex					0.647
	Male	12 (26%)	117 (28%)	3 (15%)	
	Female	35 (74%)	301 (72%)	17 (85%)	
Subtype					0.012
	Classic	24 (51%)	299 (72%)	16 (80%)	
	Follicular variant	20 (43%)	79 (19%)	2 (10%)	
	Other variants	3 (6%)	40 (10%)	2 (10%)	
Tumor size (cm)		2.7 (2.0–4.3)	2.5 (1.5–4.0)	2.4 (2.0–3.3)	0.266
Extrathyroidal extension					0.068
	None	39 (83%)	280 (67%)	13 (65%)	
	Minimal	5 (11%)	123 (29%)	6 (30%)	
	Advanced	3 (6%)	15 (4%)	1 (5%)	
Lymph node metastasis					0.072
	N0/NX	29 (62%)	229 (55%)	7 (35%)	
	N1	18 (38%)	189 (45%)	13 (65%)	
Average ploidy		1.99 (1.96–1.99)	2.00 (1.96–2.00)	2.00 (2.00–2.00)	0.002

Data are presented as frequency (percentage) or median (interquartile range).
